# Temporal and geographical variations in musculoskeletal imaging: a register-based study in Norway with focus on potential low-value imaging

**DOI:** 10.1007/s43999-025-00077-x

**Published:** 2025-11-04

**Authors:** Ingrid Øfsti Brandsæter, Jan Porthun, Eivind Richter Andersen, Bjørn Morten Hofmann, Elin Kjelle

**Affiliations:** 1 Department of Health Sciences Gjøvik at The Norwegian University of Science and Technology (NTNU), NTNU Gjøvik, PB 191, Gjøvik, 2802 Norway; 2 Centre for Medical Ethics at The University of Oslo, PB 1130, Blindern, Oslo, 0318 Norway

**Keywords:** Diagnostic imaging, Geographical variation, Low-value imaging, Musculoskeletal imaging

## Abstract

**Background:**

Musculoskeletal (MSK) issues generate extensive use of diagnostic imaging. Geographical variations in imaging are of interest as they can indicate under- and overutilisation. Low-value imaging is defined as examinations where evidence suggests it confers no or very little benefit on patients.

**Purpose:**

To assess the temporal and geographical variations in MSK imaging with special attention to two potential low-value MSK examinations, i.e. MRI of knee and MRI of lower back, in Norway from 2013 to 2022.

**Materials and methods:**

Both inpatient and outpatient examinations from 2013 to 2022 were included. Data on outpatients was collected from the Norwegian Health Economics Administration, while inpatient data was gathered directly from strategically selected hospitals in Norway. Missing inpatient data was extrapolated based on population and outpatient data for each hospital trust. The rates are adjusted for age and sex to the European Standard Population.

**Results:**

Yearly, 1,884,614 MSK examinations were conducted in Norway. I.e. 3,586 examinations per 10,000 inhabitants. The use of MSK imaging decreased by 6.8% during the study period. The geographical variations were highest for the use of the potential low-value MSK examinations, where the region with the highest use conducted over twice as many examinations than the region with the lowest use.

**Conclusion:**

The temporal variations in the use of MSK examinations was small. However, there were substantial geographical variations between the hospital trusts, and modest variations between the regional health trusts. Reducing low-value examinations could free up resources for examinations with high value.

**Supplementary Information:**

The online version contains supplementary material available at 10.1007/s43999-025-00077-x.

## Introduction

The use of diagnostic imaging is increasing globally [1], with well-documented geographical variations in the USA and Europe [2–6] and outpatient diagnostic imaging in Norway [7–12]. These variations may be due to differences in demographics and access to imaging, however, Norway is a homogeneous country regarding morbidity and the need for health care services compared to other European countries [13, 14]. The geographical variations can therefore be unwarranted and be a result of over- and under-utilisation of diagnostic imaging. This is problematic because unwarranted geographical variations are linked to ethical issues, such as injustice, harm by both under- and overutilisation, and a lack of beneficence [15]. These variations threaten the principle of equitable access to health services, an important part of the Norwegian health service [10, 16].

Overutilisation can be an indicator of low-value examinations [17], which is defined in terms of low-value care as “an intervention where evidence suggests it confers no or very little benefit on patients, or risk of harm exceeds likely benefit, or, more broadly, the added costs of the intervention do not provide proportional added benefits” [17]. Between 20 and 50% of all imaging internationally is estimated to be low-value examinations [18, 19]. These examinations have several potential negative consequences for the patients – for instance, radiation exposure, false test results and incidental findings of no clinical relevance [20–23]. In addition, low-value examinations occupy resources and contribute to increasing costs and waiting times for high-value imaging [24].

People with atraumatic pain in the musculoskeletal (MSK) system are a patient group with extensive use of diagnostic imaging, and geographical variations are also seen for this patient group [4, 5, 25]. Several MSK examinations are identified as low value for specific patient groups or clinical problems [26, 27]. Two MSK examinations, magnetic resonance imaging (MRI) of the knee and the lower back [28], are included in the Norwegian Choosing Wisely campaign – an initiative which aims to reduce the use of low-value imaging [27] (Table 1). Other studies from Norway show that more than 58% of Knee MRIs and 36% of Spine MRIs are low-value examinations [29, 30].

**Table 1:** The two recommendations from the choosing wisely initiative on MSK imaging

Choosing wisely recommendations
Avoid diagnostic imaging for low back pain without red flags. Examples of red flags are fever or other signs of infection, history of injury or recent spinal puncture accompanying general symptoms. If uncomplicated disc herniation or uncomplicated spinal stenosis is suspected, imaging is only indicated after 4–6 weeks of conservative treatment and if surgery is being considered.
Avoid advanced diagnostic imaging for anterior knee pain if the patient does not have hydrops or locking or has tried physical treatment without improvement. Conservative treatment is the first choice. In case of poor recovery, swelling, clicking or locking, an MRI can be useful.

MRI: Magnetic resonance imaging, [28]

While unwarranted geographical and temporal variations can indicate underuse and overuse of diagnostic imaging, low-value imaging is one reason for overutilisation which can generate unnecessary waiting times and shortfalls [31]. Little is known about the use of and variations in potential low-value MSK imaging in Norway. Accordingly, this study aimed to assess the temporal and geographical variations for inpatient and outpatient diagnostic MSK imaging in Norway from 2013 to 2022 and to estimate the extent of and variations in potential low-value imaging. The research questions of this study were:

1. How did MSK imaging vary over time and for different imaging modalities in Norway from 2013 to 2022?
2. How did the use of MSK imaging vary between regions in Norway from 2013 to 2022?
3. How did the extent of potential low-value MSK imaging vary in Norway from 2013 to 2022?

## Materials and methods

### Setting

Norway is a long and narrow country consisting of remote, rural, and urban areas. In the most remote areas, travel time to hospitals and imaging centres may be several hours. One key principle for the Norwegian health service is equitable access to health services, regardless of where people live [16]. Compared to the countries in the European Economic Area (EEA), Norway has the highest coverage of physicians and employees in the health care services per capita [13, 16].The public healthcare system is divided into primary and specialist healthcare. Diagnostic imaging is part of the latter [16]. The specialist healthcare system is organised into four regional health authorities (RHAs): Western, Central, Northern, and South-Eastern Norway RHA (see Supplementary File 1). The RHAs consist of in total of 19 hospital trusts (HTs). The HTs have the responsibility for the hospital care [32]. The RHAs can also have contracts with not-for-profit hospitals to conduct hospital care, including diagnostic imaging [16]. Hereafter, the HTs and not-for-profit hospitals will be called hospitals. All Norwegian residents have universal health service coverage through general taxation, thus, patients only pay a co-pay element for each examination, €24 in 2024 [16].

Oslo, the capital of Norway, is located within the South-Eastern Norway RHA, which has the largest population (56% in 2022) compared with the other RHAs [33]. Additionally, Oslo is home to both the national hospital, Rikshospitalet University Hospital, and the national cancer hospital, Radiumhospitalet, which are both part of Oslo University Hospital.

Private imaging centres are also included in this study. The imaging centres have tender agreements with the RHAs with the RHAs to conduct outpatient diagnostic imaging on public terms [34]. All HTs except for Førde HT, Nord-Trøndelag HT, Finnmark HT and Helgeland HT have a private imaging centre located in their catchment area. Private imaging centres also provide out-of-pocket services and imaging paid for by private health insurance. Examinations paid out-of-pocket or by private health insurance amount to around 10% of diagnostic imaging in Norway [16].

### Musculoskeletal examinations

Diagnostic examinations are classified by the Norwegian Classification of Radiological Procedures (NCRP) codes [35]. In total, over 1,000 NCRP codes exist, covering all imaging modalities, including MRI, conventional radiography (CR), computed tomography (CT), ultrasound (US), and nuclear medicine (NM) [35]. In 2016, all NCRP codes were changed, some were merged, and others were divided. All NCRP codes were therefore converted to the 2016 version in this study. As the data did not include the indication for imaging, it was not possible to distinguish whether an NCRP code was used for MSK indications or other conditions. Hence, all NCRP codes relevant to MSK examinations were compiled for this study, resulting in a list of 98 NCRP codes (Supplementary File 2).

For research question 3, the present study focuses on the two examinations: Lower Back MRI and Knee MRI (NCRP codes SNA0GG and SNG0AG). The two examinations are identified as low-value for specific patient groups or indications (Table 1), and an overuse of these examinations can indicate that unnecessary or low-value imaging is conducted. As we do not know the indication or outcome of the examination, one cannot calculate the number of low-value imaging, thus, these examinations are presented as ‘potential’ low-value imaging.

### Data collection

Outpatient data were collected from the Norwegian Health Economics Administration. Further, as there is no central registry of performed inpatient examinations, the inpatient data was collected directly from a strategic selection of 15 hospitals in Norway, including all examinations in one of the RHAs. In total, 90.5% of inpatient and outpatient examinations, distributed across all RHAs, were collected. The missing inpatient data (9.5% of all examinations and 34.7% of the inpatient data) was extrapolated (further explained in Statistical Analysis).

The dataset included information about the patient’s age and sex, examination name, NCRP code, imaging modality, name of hospital/imaging centre, and status as inpatient or outpatient.

The RHAs and HTs’ catchment areas were used to define the geographical regions used in the analysis. Examinations conducted in the private imaging centres and not-for-profit are included in the respective HT/RHA in which catchment area they are located. For instance, the number of examinations conducted in Western Norway RHA includes examinations from public and not-for-profit hospitals and the imaging centres located in this area. The data do not provide information about where the patient lived. If a patient living in Bergen is referred to imaging in Oslo, the examination is a part of the Oslo rate.

Data on the Norwegian population on January 1st per year was provided by Statistics Norway [33].

### Statistical analysis

To assess the temporal and geographical variations in Norway, SPSS Statistics, version 28 (IBM Corp.) and Microsoft Excel version 2305 were used for descriptive statistics.

Rates per 10,000 inhabitants are calculated by dividing the number of examinations in each 10-year age group, for both sexes, by the respective population for that group and region. These rates are then age- and sex-standardised using direct adjustment based on the European Standard Population [36].

The annual average is used to present regional rates, in addition, the median and the 25th and 75th percentiles are used to describe the ratio between regions.

For temporal variations, the average of the earliest 2 annual rates (2013 and 2014) is compared to the latest 2 annual rates (2021 and 2022), to stabilise the comparison.

Due to resources, inpatient data was collected from a representative selection of hospitals. In total, inpatient examinations were missing from 11 hospitals for the whole study period, and two hospitals missed data for part of the study period.

To estimate the missing inpatient examinations, an extrapolation was conducted. In the collected inpatient data, all RHAs were represented. One of the four RHA provided a complete data set that included all inpatient data from all HTs within the RHA. This complete dataset was included in the analysis; thus, by using a full data set of both in-and outpatients, we could calculate the distribution of inpatient and outpatient examinations per inhabitant. An extrapolation for the missing hospitals was made based on the respective hospitals’ use of outpatient examination, the catchment area inhabitant number and population distribution. Hospitals with similar characteristics (as size of hospital and catchment area, and whether the hospital are located in rural or urban areas) were paired to reduce potential skewness. In total, 32% of the inpatient examinations were extrapolated.

As all outpatient examinations were collected, the extrapolation was only conducted for inpatient examinations. In the present study, only a small percentage (13%) of the examinations are inpatient examinations and almost 70% of these are collected from the hospitals. Thus, the missing values will have an insignificant effect on the results.

## Results

### Temporal variations in musculoskeletal imaging

The 98 MSK NCRP codes yielded 1,884,614 MSK examinations yearly in Norway, i.e. 3,568 per 10,000 inhabitants. More females (56%) than males (44%) had an MSK examination during the study period. Table 2 shows the distribution between the imaging modalities used for MSK examinations. CR is the imaging modality most used, followed by MRI. No NM codes were identified as MSK examinations.

**Table 2:** Average annual use of musculoskeletal examinations per 10,000 inhabitants, standardised by European standard population

Imaging modality	*N* annual average (± SD)
CR	2,573 (161)
MRI	817 (32)
CT	120 (11)
US	77 (3)
Total	3,586 (163)

SD: Standard deviation, CR: Conventional radiography, MRI: Magnetic resonance imaging, CT: Computed tomography, US: Ultrasound

**Fig. 1:**
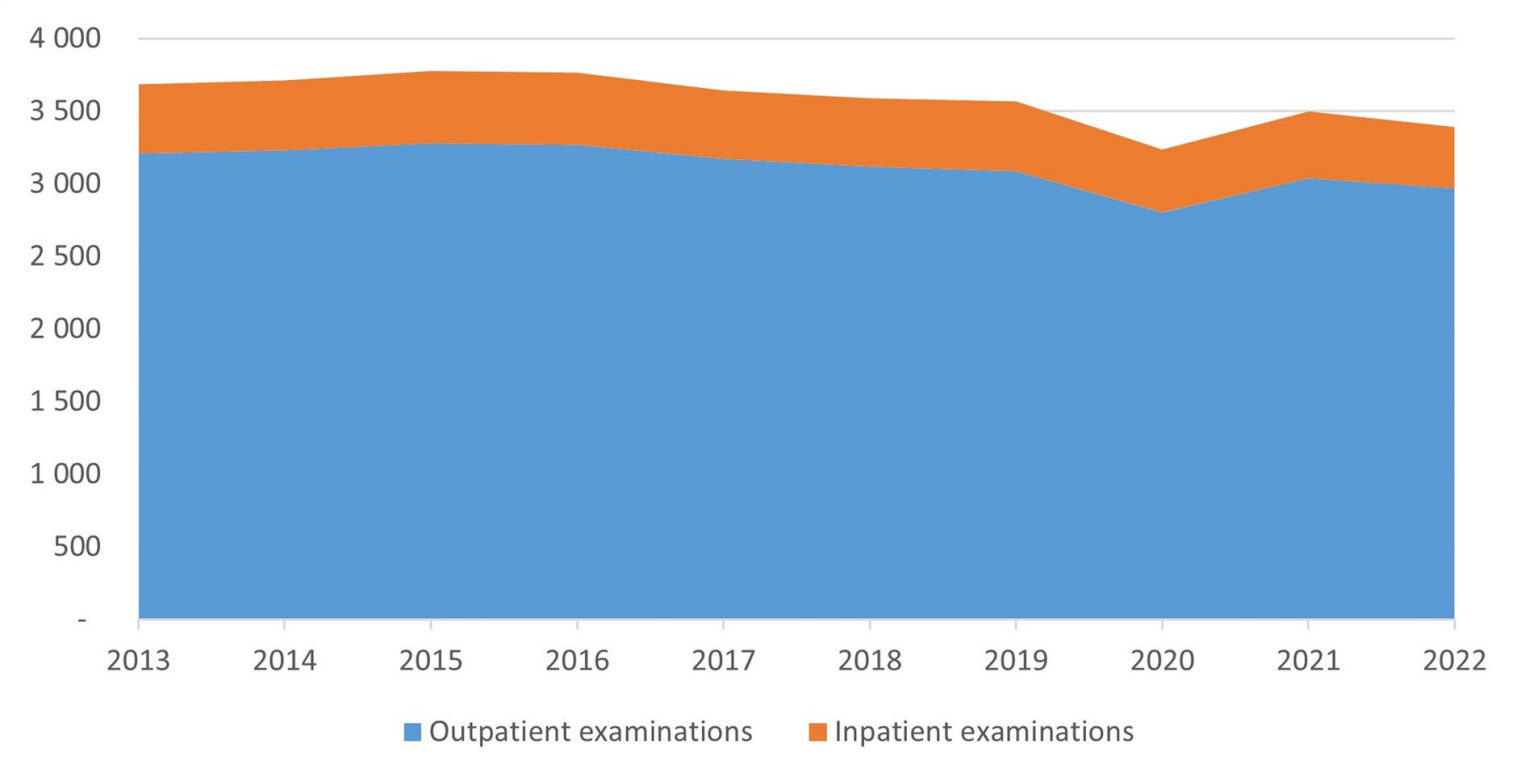
Temporal variations in musculoskeletal examinations per 10,000 inhabitants in Norway between 2013 and 2022. Divided into inpatient and outpatient examinations

From 2013 to 2022, the total rate of MSK imaging decreased from 3,686 to 3,392 examinations per 10,000 inhabitants. Comparing the mean of the earliest annual rates (2013 and 2014) to the latest annual rates (2021 and 2022), there was a 6.8% decrease during the study period. There was a clear reduction in the use in 2020 (3,237 examinations), mainly due to SARS-CoV-2, whilst in 2021 the use of MSK imaging was on the same level as in 2019. On average, 13% of the examinations were inpatients in hospitals, while 58% were outpatients in hospitals and 29% were outpatients in private centres. The temporal variations for inpatient and outpatient examinations are shown in Fig. 1.

### Geographical variations in musculoskeletal imaging

The median use of MSK imaging across the four regions was 3,559 examinations per 10,000 inhabitants annually (with a 25th percentile and 75th percentile at 3,441 and 3,708, respectively, yielding a ratio of 1.08). Overall, the average annual use per inhabitant was highest in Central Norway RHA and lowest in Western Norway RHA.

**Fig. 2:**
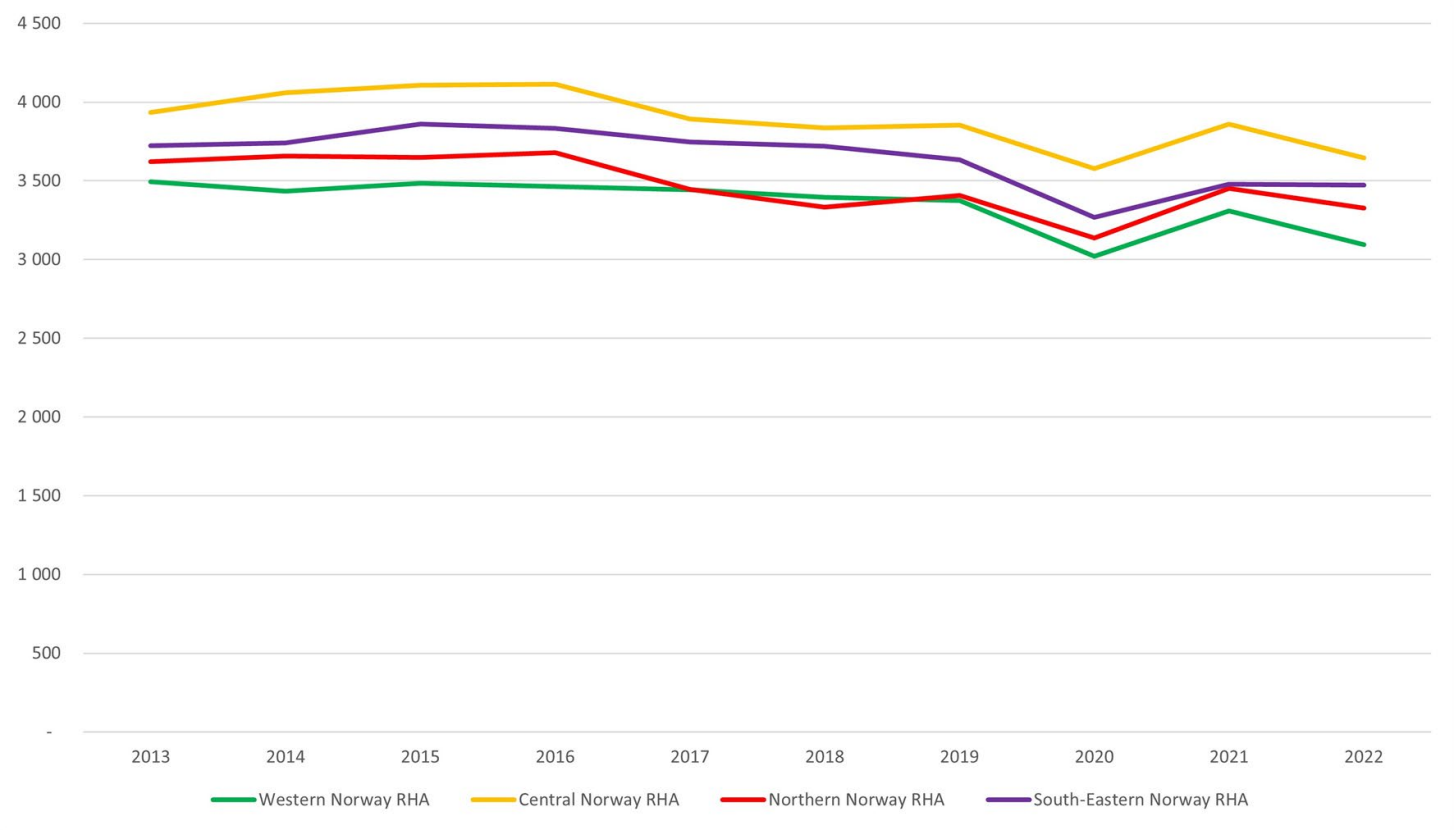
Temporal variations in musculoskeletal examinations per 10,000 inhabitants in the four geographical areas in Norway between 2013 and 2022

The number of examinations per 10,000 inhabitants decreased during the study period in all four RHAs. Comparing the mean of the earliest 2 annual rates to the mean of the latest 2 annual rates, Western Norway RHA had the biggest decrease by 7.6% (from 3,464 to 3,201). South-Eastern Norway RHA and Northern Norway RHA, both, had a decrease of 6.8% (from 3,732 to 3,477 and from 3,639 to 3,390, respectively), and Central Norway RHA decreased by 6.1% (from 3,997 to 3,753). Figure 2 shows the temporal variations in MSK imaging per RHA.

There were small variations in the use of the various imaging modalities. Overall, Central Norway RHA had the highest use of MSK imaging, accounting for 27% of the total use. South-Eastern Norway RHA comprised 25% of MSK imaging, while Northern Norway RHA and Western Norway RHA accounted for 24% and 23%, respectively.

**Fig. 3:**
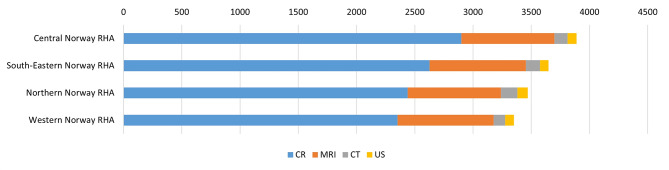
Geographical variations in the use of MSK imaging modalities in the four RHAs in Norway (per 10,000 inhabitants). Numbers presented as average annual examinations in Norway from 2013 to 2022. MRI: magnetic resonance imaging, CR: conventional radiology, CT: computer tomography, US: ultrasound

CR and MRI comprised the majority of the MSK imaging. Central Norway RHA accounted for 28% of the CR usage, followed by South-Eastern Norway RHA (25%), Northern Norway RHA (24%) and Western Norway RHA (23%). The use of MRI was similar across all four regions. Figure 3 shows the use of the various modalities for MSK imaging in the four RHAs.

### Top 20 most used musculoskeletal examinations

Table 3 presents the top 20 most used MSK examinations in 2022 per 10,000 inhabitants. CR and MRI were the only two imaging modalities on this list. Most of the examinations in the top 20 list had the lowest use in 2020, mainly due to the COVID-19 pandemic.

Comparing the earliest 2 annual rates to the latest 2 annual rates, the use of CR of the pelvis shows a small increase by 2%, and the use of CR Clavicle was stable during the study period. The other CR examinations in the top 20 list had a reduction throughout the study period (ranging from a 4 to 36% decrease).

Most of the MRI examinations on the list increased in use during the study period.). MRI of the Hip increase the most (82%), followed by MRI Lumbosacral spine (in this paper called Lower Back MRI). However, a new code, MRI Pelvis and Part of Spine, was introduced in 2016, and the use of this new code increased steadily in the following years. As this new code can have affected the use of the other codes for MRI Pelvis and Lower Back, the codes are combined in a new row named ‘MRI Pelvic and Lower Back (combined)’ in Table 3.

**Table 3:** Top 20 most used musculoskeletal examinations in Norway in 2022 and the Temporal variation from 2013 to 2022, per 10,000 inhabitants. The right column (% change) describes the change from two first years to the two last years of the study period

Examination	2013	2014	2015	2016	2017	2018	2019	2020	2021	2022	Change %
CR Knee	339	332	332	337	327	315	317	280	309	300	− 9
CR Hip	388	376	374	369	353	337	334	293	313	301	− 20
CR Hand	304	300	313	309	303	304	304	276	296	285	− 4
CR Pelvis	275	278	290	291	284	284	287	260	284	283	2
CR Foot	289	298	294	293	288	289	284	248	267	256	− 11
CR Wrist	244	234	236	237	228	228	221	203	220	204	− 11
CR Ankle	224	223	228	224	219	221	217	195	212	205	-7
CR Shoulder	190	183	182	176	169	166	156	140	152	145	− 21
CR Lumbosacral spine	134	134	140	128	114	103	102	90	89	83	− 36
CR Elbow joint	75	74	73	74	72	69	71	64	69	64	− 11
CR Leg	61	59	57	57	56	56	53	48	53	49	− 15
CR Clavicle	32	32	32	33	32	34	32	30	33	31	0
MRI Knee	161	164	161	171	170	161	166	150	160	150	− 5
MRI Lumbosacral spine	188	201	210	198	172	166	165	154	155	146	− 23
MRI Shoulder	108	111	113	116	120	118	116	109	119	111	5
MRI Pelvis	51	55	62	68	47	49	52	47	51	52	− 3
MRI Hip	23	26	31	46	55	39	40	40	44	44	82
MRI Pelvis and part of the Spine	0	0	0	0	28	35	38	39	51	49	60^a^
MRI Ankle joint	26	30	33	34	37	39	41	38	42	40	45
MRI Foot	26	29	33	35	39	40	40	36	41	38	44
MRI Pelvic and Lower Back (combined)^b^	239	256	273	265	247	249	255	240	257	247	2

^a^ % Change from 2017–2022

^b^ Combined use of the three examinations: MRI Pelvis, MRI Lower Back, and ‘MRI Pelvis and Part of Spine’

CR: conventional radiology, MRI: magnetic resonance imaging

### Variations in potential low-value musculoskeletal imaging

Knee MRI and Lower Back MRI are well documented to be of low value for various conditions. These examinations were the two most used MRI examinations in MSK imaging, which constitutes to approximately 10% of the total MSK examination.

**Fig. 4:**
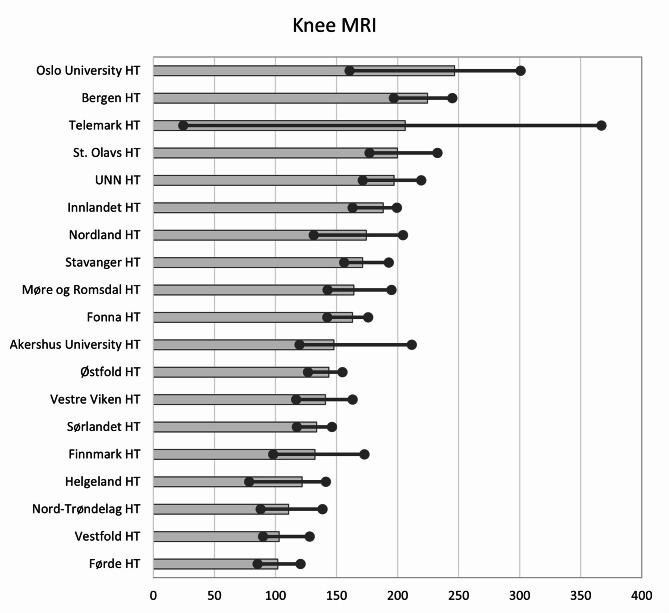
Variation in average number of Knee MRIs per 10,000 inhabitants for 2013–2022 for the hospital trust catchment areas. Dots represent minimum and maximum during the years 2013–2022. HT: Hospital Trust, UNN: University hospital of Northern Norway

The use of Knee MRIs decreased by 5% (from 161 to 150) throughout the study period. Seventy-seven per cent of Knee MRIs were conducted in private imaging centres. Across the HTs’, the median number of Knee MRI per year was 163 (with the 25th percentile 133 and 75th percentile at 193, yielding a ratio of 1.45). Oslo University HT had the highest use per inhabitant (247 annual examinations per 10,000 inhabitants) 2,4 times more examinations than Førde HT (102 annual examinations per 10,000 inhabitants), which had the lowest use per inhabitant. The regional variations for Knee MRI are shown in Fig. 4.

**Fig. 5:**
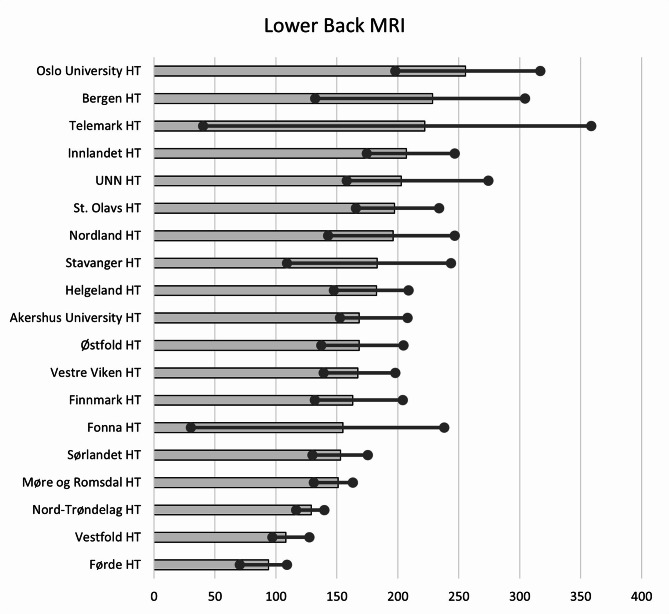
Variation in average number of Lower Back MRI per 10,000 inhabitants for 2013–2022 for the hospital trust catchment areas. Dots represent minimum and maximum during the years 2013–2022. HT: Hospital Trust, UNN: University hospital of Northern Norway

The number of Lower Back MRIs (MRI Lumbosacral spine) declined by 23% (from 188 to 146) during the study period. Seventy-one per cent of the Lower Back MRIs were performed in private imaging centres. Across the HTs, the median number of Lower Back MRI annually was 168 examinations per 10,000 inhabitants with the 25th and 75th percentiles at 154 and 200, respectively, giving a ratio of 1.30. In the 19 HT’s, the average number of Lower Back MRIs varied from 255 (Oslo University HT) to 94 (Førde) HT examinations per 10,000 inhabitants. The regional variations for Lower Back MRI are shown in Fig. 5.

**Fig. 6:**
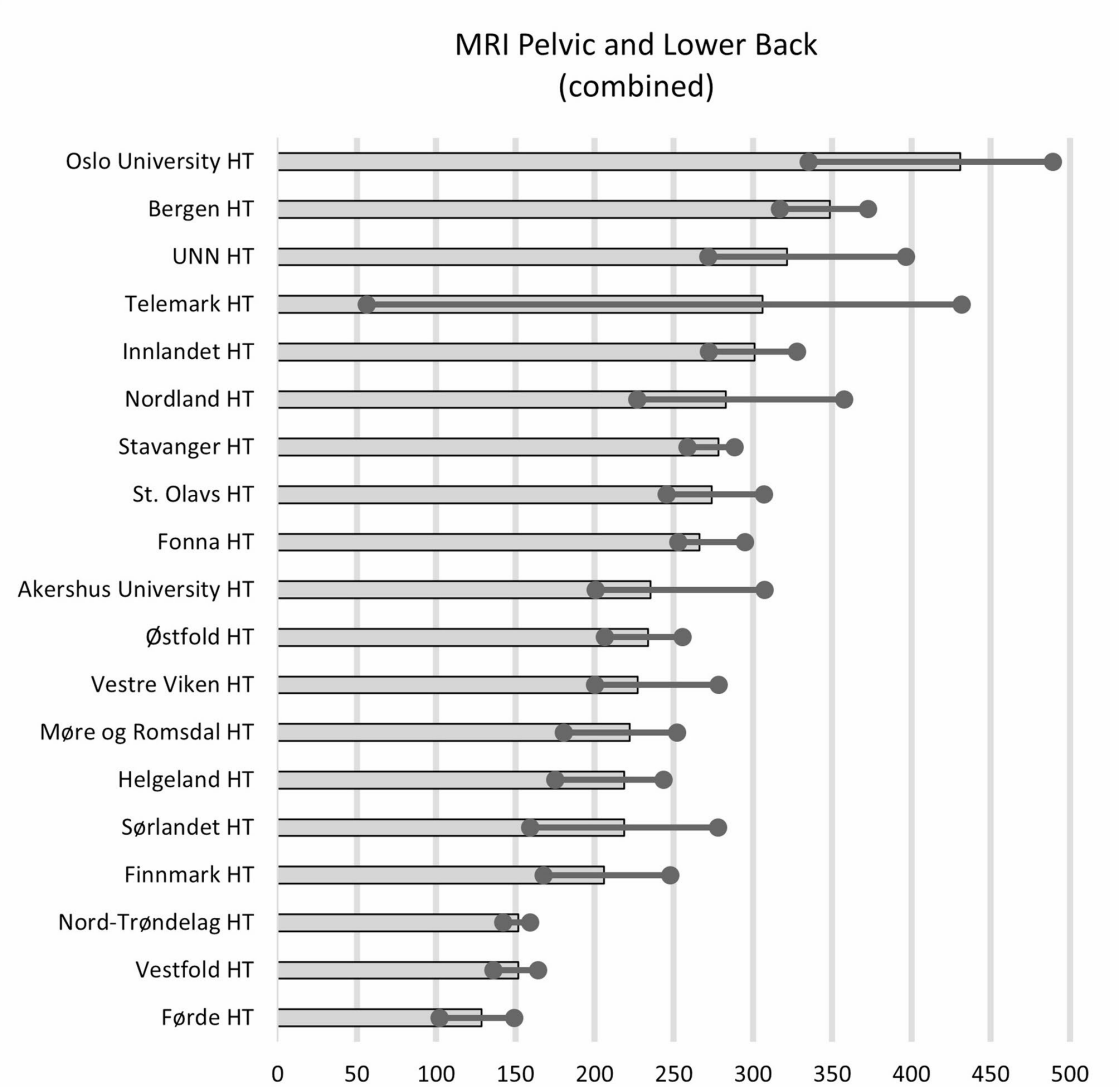
Variation in average number of the combined codes for MRI Pelvic and Lower Back per 10,000 inhabitants for 2013–2022 for the hospital trust catchment areas. Dots represent minimum and maximum during the years 2013–2022. HT: Hospital Trust, UNN: University hospital of Northern Norway

The new code, MRI Pelvis and Part of Spine, was introduced in 2016, and can have affected the use of the other codes for MRI Pelvis and MRI Lower Back. Across the HTs, the median number of Lower Back MRI annually was 235 examinations per 10,000 inhabitants, with the 25th and 75th percentiles at 219 and 292, respectively, which gives a ratio of 1.34. The geographical variations of the combined code (combined use of the three examinations: MRI Pelvis, MRI Lower Back and ‘MRI Pelvis and Part of Spine’), are bigger compared to the use of Lower Back MRI alone. Oslo University HT had 3,4 times higher use than Førde HT (431 and 129 examinations per 10,000 inhabitants, respectively) (Fig. 6).

## Discussion

The aim of the study was to assess the variations in Norway both over time and between geographical regions in the use of MSK imaging and for two potential low-value MSK examinations. The study does not measure the number of low-value imaging, but the geographical variations can indicate that low-value imaging is conducted in Norway.

The temporal variations and the geographical variations between the RHAs were relatively small, with a ratio of the 75th to 25th percentiles of 1.08, while the differences between the HTs were moderate with a ratio of 1.30–1.45. The use of MSK imaging was stable throughout the period, except for a decrease in 2020. However, in 2021, the number of examinations was back to the same level as in 2019. The same tendencies were seen for the two potential low-value MSK examinations. These results align with other research that saw a decrease in the use of diagnostic imaging in the first year of the COVID-19 pandemic [37, 38].

There was a decrease in the use of MSK imaging from 2015 to 2022 per inhabitant. However, the Norwegian population increased during this period [33]. Thus, the total use of diagnostic imaging was rather steady, and the use of resources may be stable throughout the study period.

There were substantial geographical variations between the various HT, and variations of two times or more are considered clinically significant as they impose substantial opportunity costs. Several international studies have found geographical variations in diagnostic imaging [2, 4, 39, 40], and our results are in line with previous Norwegian studies of geographical variations [10, 12] and outpatient MSK examinations [9, 11, 41]. The present study adds to this by providing an extension of inpatient examinations.

In rural areas of Norway, the travel time to hospitals and imaging centres may be several hours, whilst in urban areas, the availability of diagnostic imaging is higher [34]. Availability is documented to be a driver for low-value imaging in Norway [42, 43] and can be one explanation for the geographical variations of diagnostic imaging. For instance, Fig. 5 shows that the four areas without a private imaging centre located within the HT’s catchment area are among the five HTs with lowest use of Knee MRI per inhabitant. However, these four areas are more spread regarding the use of Lower Back MRI, where Helgeland has the same level of use as the mean of Norway. Travel time to health care services is also seen as a reason for geographical variations in other countries, for instance, the use of radiotherapy in Italy [44]. On the other hand, another study from Norway found no difference in the use of MRI concerning travel distance [45]. This demonstrates that people in rural areas in Norway are willing to travel for health care services [34, 45]. Moreover, the supply of imaging equipment could be a reason for regional differences, as it affects the availability of imaging. However, a study by Hofmann et al. [12] found that Region West Norway overall had higher rates of MRI examinations per 10,000 inhabitants than Region South-East, which had more than twice as many MRI machines. Thus, indicates that the supply of equipment does not necessarily affect the rate of examinations.

Norway is a homogeneous country concerning morbidity and the use of health care services [14]. Thus, there must be other and potentially unwarranted reasons for these variations. One reason might be an overuse in the regions with the highest use and underuse in other areas. Another reason for variations might be different routines in the use of the NCRP codes [7]. These results do not provide an answer concerning the right level of examinations; however, as MSK constitutes a great proportion of the total use of diagnostic imaging, it is important to have knowledge about the different levels, to try to find the right level of use.

The MSK examinations assessed in this study, Knee MRI and Lower Back MRI, are well-documented to be of low value for specific indications [46–49]. The data in the present study do not include the examinations’ indication or outcome. Although earlier Norwegian studies have found that between 58% and 85% of the patients have a Knee MRI [29], and 36% of MRIs of the spine [30] had no relevant examinations or diagnoses from the public healthcare services six months before or after the MRI examination, which indicates that the examinations were unnecessary. Applying these estimates of low-value imaging, 54,693 (58%) Knee MRIs and 31,433 (36%) Lower Back MRIs were of low value in one year.

Further, Knee MRI and Lower Back MRI were the two most used MRI MSK examinations (Table 3), which indicate a high utilisation. One study has estimated the cost of low-value Knee MRI in Norway to cost between € 6,7 and 9,8 million each year [29] so even a reduction of 5% would free up a lot of resources for high-value examinations.

In 2016, a new set of NCRP codes was made, which could have affected the code use. For instance, the code for ‘MRI Pelvis and Part of the Spine’ presented in Table 3 had no examinations from 2013 to 2015. Table 3 shows that as the number of MRIs Pelvis and Part of Spine increases, the number of Lower Back MRIs (MRI Lumbosacral spine) decreases. Combining these three MRI codes for the Pelvis, Pelvis and Part of Spine and MRI Lower Back, the number of examinations is stable during the study period. This might also be the case for other examinations. Shifts in clinical practice over time could also account for certain time-related variations. For instance, the Top 20 list showed that CR of the hip had a reduction on 20%, while MRI of the hip increased by 80%, which may imply a shift from CR to MRI for hip imaging.

One strength of this study is that the dataset includes both inpatient and outpatient data. Even though we did not have data from all HT, extrapolation made it possible to estimate the use in all hospitals.

One limitation of the study is that the data set did not include information on where the patients lived, only where the examination was conducted. This may result in skewness, as some patients may travel to another HT than their local hospital to have the examination, for instance by travelling to a specialist hospital or an imaging centre located in another HT’s catchment area. The hospitals within one RHA may have various areas of expertise and thus different patient populations. By using the RHAs as comparable regions, these differences will be small, as the four RHAs have more similar patient populations. The only exception is the specialist hospitals in the South-Eastern Norway RHA.

Another limitation in the study is the use of extrapolation of inpatient imaging. However, only a small percentage (13%) of the MSK examinations are inpatient examinations and of these, almost 70% of these are collected directly from the hospitals. Thus, the missing values will have an insignificant effect on the results.

The data only includes the code for the examination and not the indication. Therefore, some examinations defined as MSK examinations were possibly not conducted on an MSK issue. For instance, the code for MRI of the hip can also be used for neurological issues. However, we have included these codes in the MSK list, as this is defined as an MSK examination in other literature [26, 27]. Another limitation is that the dataset is not complete; for example, some US examinations are sometimes conducted and registered in hospital units other than the imaging department. In addition, the dataset does not include examinations paid out-of-pocket or paid by private health insurance. Approximately 10% of the population has private health insurance [16].

In conclusion, there are small temporal variations in the use of MSK examinations, the geographical variations across the HTs are substantial, and modest across the RHAs. Correspondingly, potential low-value MSK examinations also vary between the geographical areas and over time. Low-value imaging has potential negative consequences and takes up resources. Reducing the use of these examinations could free up resources for examinations with high value. More research on variation in the use of imaging and low-value services is warranted. Future research should aim to find the right level of MSK imaging utilisation and implement measures to reduce the use of low-value MSK imaging.

## Electronic Supplementary Material

Below is the link to the electronic supplementary material.

Supplementary File 1

Supplementary File 2

## Data Availability

The datasets generated and analysed during the current study are not publicly available due to regulation by the REC. However, aggregated data are available from the corresponding author on reasonable request.
