# Supplementary material for: Temporal and geographical variations in musculoskeletal imaging: a register-based study in Norway with focus on potential low-value imaging
Source: Res Health Serv Reg. 2025 Nov 4;4:17. doi: 10.1007/s43999-025-00077-x (PMC12583358; doi:10.1007/s43999-025-00077-x)
Supplement: Supplementary file 1 [file 43999_2025_77_MOESM1_ESM.pdf]

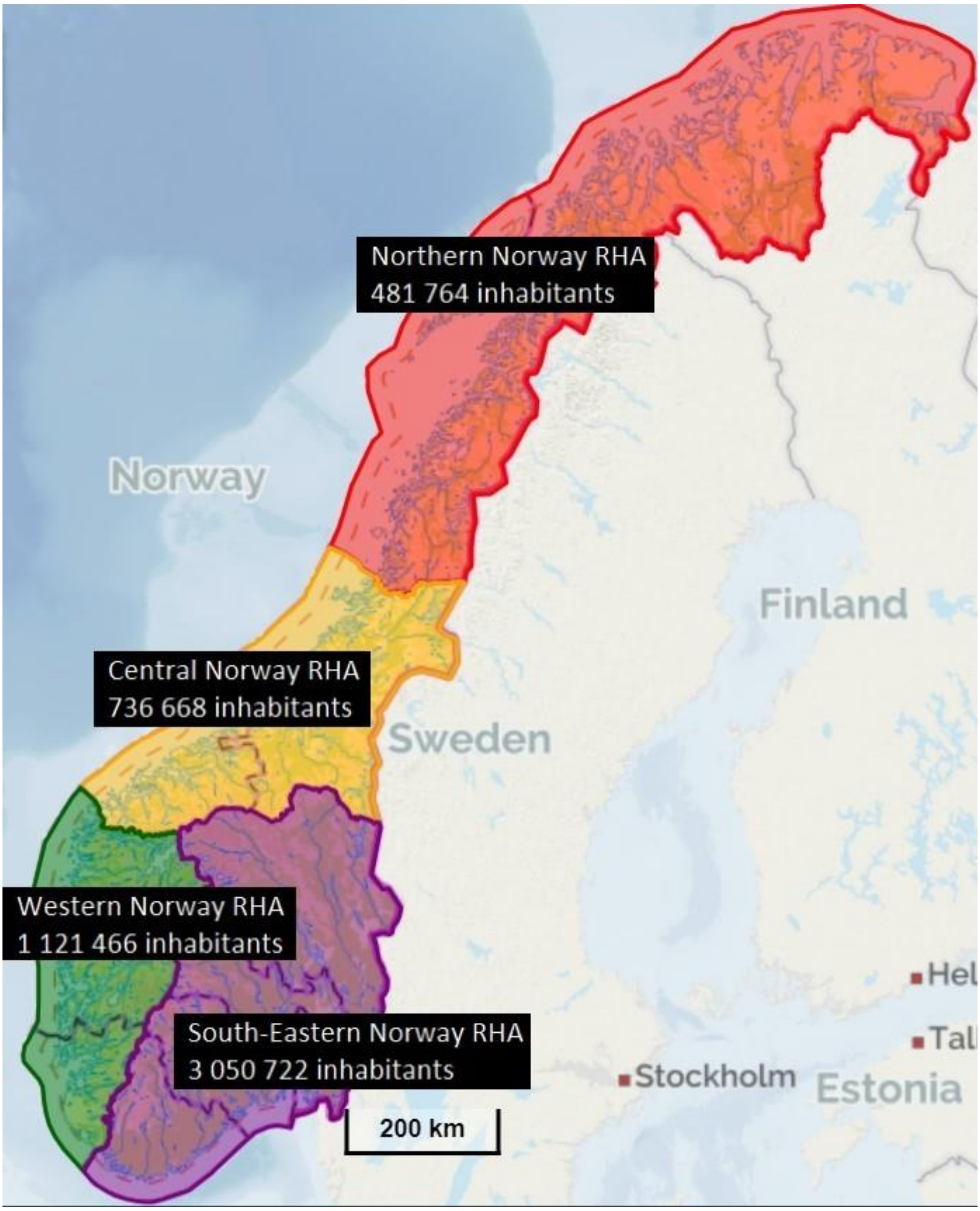

The four RHAs in Norway: Western, Central, Northern and South-Eastern Norway Regional Health Authorities. Inhabitant number 01.01.2021. RHA: Regional Health Authority.

Article Title: Temporal and Geographical Variations in Musculoskeletal Imaging: a Registry Based Study in Norway with Focus on Potential Low-Value Imaging

Journal name: Research in Health Services & Regions, Methods, Results, Implementation

Author names: Ingrid Øfsti Brandsæter, MSc [1] ingrid.o.brandsater@ntnu.no (Corresponding author), Jan Porthun[1], Eivind Richter Andersen, MSc [1], Bjørn Morten Hofmann, PhD [1,2] , Elin Kjelle, PhD [1]

[1]Department of Health Sciences Gjøvik at the Norwegian University of Science and Technology (NTNU), Norway

Address: NTNU Gjøvik, PB 191, 2802 Gjøvik, Norway

[2]Centre for Medical Ethics at the University of Oslo, Norway

Address: Centre for Medical Ethics, PB 1130, Blindern, 0318 Oslo
