# Supplementary material for: Temporal and geographical variations in musculoskeletal imaging: a register-based study in Norway with focus on potential low-value imaging
Source: Res Health Serv Reg. 2025 Nov 4;4:17. doi: 10.1007/s43999-025-00077-x (PMC12583358; doi:10.1007/s43999-025-00077-x)
Supplement: Supplementary file 2 [file 43999_2025_77_MOESM2_ESM.pdf]

Supplementary File 2: NCRP codes relevant for musculoskeletal examinations

In Temporal and Geographical Variations in Musculoskeletal Imaging: a Registry Based Study in Norway with Focus on Potential Low-Value Imaging

Journal: Research in Health Services & Regions, Methods, Results, Implementation

Authors: Ingrid Øfsti Brandsæter, MSc [1] ingrid.o.brandsater@ntnu.no (Corresponding author), Jan Porthun[1], Eivind Richter Andersen, MSc [1], Bjørn Morten Hofmann, PhD [1,2] , Elin Kjelle, PhD [1]

[1]Department of Health Sciences Gjøvik at the Norwegian University of Science and Technology (NTNU), Norway. Address: NTNU Gjøvik, PB 191, 2802 Gjøvik, Norway

[2]Centre for Medical Ethics at the University of Oslo, Norway. Address: Centre for Medical Ethics, PB 1130, Blindern, 0318 Oslo

**NCRP**	**Examination**
SNC0AA	CR Elbow
SNH0BA	CR Ankle
SNE0AA	CR Pelvis
SNB0CA	CR Clavicula
SGA0BA	CR Costae
SNH0AA	CR Foot
SNF0AA	CR Hip
SND0BA	CR Hand
SND0AA	CR Wrist
SNG0AA	CR Knee
SNG0BA	CR Leg
SAB0GA	CR Lumbar myelography
SNA0GA	CR lumbosacral spine
SNF0BA	CR Thigh
SNB0AA	CR Upper arm
SNI0AA	CR Upper extremity
SNE0CA	CR Sacrum with coccyx
SNB0BA	CR Shoulder
SNB0EA	CR Shoulder blade
SGA0AA	CR Sternum
SNA0FA	CR Thoracic and lumbar spine
SNA0BA	CR Thoracic spine
SNA0JA	CR Thoracic and lumbosacral spine
SNA0KA	CR Total spine

SNJ0AA	CR Lower extremity
SNC0BA	CR Underarm
SNC0AD	CT Elbow
SNH0BD	CT Ankle
SSE0AD	CT Pelvis
SSM0AD	CT Pelvis and lower extremity
SNA0ED	CT Cervikal and thoracic spine
SNH0AD	CT Foot
SNF0AD	CT Hip
SND0BD	CT Hand and fingers
SND0AD	CT Wrist
SNE0BD	CT Sacroiliac joint
SNG0AD	CT Knee
SNG0BD	CT Leg
SNA0GD	CT Lumbosacral spine
SNF0BD	CT Thigh
SNB0AD	CT Upper arm
SNI0AD	CT Upper extremity
SNB0BD	CT Shoulder
SGA0AD	CT Sternum
SNA0BD	CT Thoracic spine
SNA0JD	CT Thoracic and lumbar spine
SNA0KD	CT Total spine
SNC0BD	CT Underarm
SNJ0AD	CT Lower extremity
SNJ0BD	CT Lower extremity measurement
SNC0AG	MRI Elbow
SNH0BG	MRI Ankle
SSE0AG	MRI Pelvis
SNA0TG	MRI Pelvis and part of spine
SSM0AG	MRI Pelvis and lower extremity
SNA0PG	MRI Caput, spine and upper extremity
SNA0SG	MRI Cervikal and lumbosacral spine
SNA0EG	MRI Cervikal and thoracic spine
SNA0HG	MRI Cervikal, thoracic and lumbar spine
SNH0AG	MRI Foot
SNF0AG	MRI Hip
SND0BG	MRI Hand and fingers
SND0AG	MRI Wrist
SNE0BG	MRI Sacroiliac joint
SNG0AG	MRI Knee
SNG0BG	MRI Leg

SNA0GG	MRI Lumbosacral spine
SNF0BG	MRI Thigh
SNB0AG	MRI Upper arm
SNI0AG	MRI Upper extremity
SNB0BG	MRI Shoulder
SGA0AG	MRI Sternum
SNA0FG	MRI Thoracic and lumbar spine
SNA0BG	MRI Thoracic spine
SNA0JG	MRI Thoracic and lumbosacral spine
SNA0KG	MRI Total spine
SNA0MG	MRI Total spine and pelvis
SNC0BG	MRI Underarm
SNJ0AG	MRI Lower extremity
SNC0AK	US Elbow
SNH0BK	US Ankle
SSE0AK	US Pelvis
SSM0AK	US Pelvis and lower extremity
SNH0AK	US Foot
SNF0AK	US Hip
SND0BK	US Hand and fingers
SND0AK	US Wrist
SNG0AK	US Knee
SNA0AK	US Spine
SNX0DK	US Joints and soft tissue, musculoskeletal system
SNX0BK	US Joints, musculoskeletal system
SNG0BK	US Leg
SNX0AK	US Groin
SNF0BK	US Thigh
SNB0AK	US Upper arm
SNB0BK	US Shoulder
SGA0AK	US Sternum
SNC0BK	US Underarm
